# JNJ-26481585 primes rhabdomyosarcoma cells for chemotherapeutics by engaging the mitochondrial pathway of apoptosis

**DOI:** 10.18632/oncotarget.6097

**Published:** 2015-10-12

**Authors:** Ulrike Heinicke, Johanna Kupka, Simone Fulda

**Affiliations:** ^1^ Institute for Experimental Cancer Research in Pediatrics, Goethe-University, Frankfurt, Germany; ^2^ German Cancer Consortium (DKTK), Heidelberg, Germany; ^3^ German Cancer Research Center (DKFZ), Heidelberg, Germany

**Keywords:** apoptosis, cell death, rhabdomyosarcoma, HDACI, mitochondria

## Abstract

Rhabdomyosarcoma (RMS) is a common soft-tissue sarcoma in childhood with a poor prognosis, highlighting the need for new treatment strategies. Here we identify a synergistic interaction of the second-generation histone deacetylase inhibitor (HDACI) JNJ-26481585 and common chemotherapeutic drugs (i.e. Doxorubicin, Etoposide, Vincristine, Cyclophosphamide and Actinomycin D) to trigger apoptosis in RMS cells. Importantly, JNJ-26481585/Doxorubicin cotreatment also significantly suppresses long-term clonogenic survival of RMS cells and tumor growth *in vivo* in a preclinical RMS model. Mechanistically, JNJ-26481585/Doxorubicin cotreatment causes upregulation of the BH3-only proteins Bim and Noxa as well as downregulation of the antiapoptotic proteins Mcl-1 and Bcl-x_L_. These changes in the ratio of pro- and antiapoptotic Bcl-2 proteins contribute to JNJ-26481585/Doxorubicin-mediated apoptosis, since knockdown of Bim or Noxa significantly inhibits cell death. Also, JNJ-26481585 and Doxorubicin cooperate to stimulate activation of Bax and Bak, which is required for JNJ-26481585/Doxorubicin-induced apoptosis, since silencing of Bax or Bak protects against apoptosis. Consistently, overexpression of Bcl-2 significantly reduces JNJ-26481585/Doxorubicin-mediated apoptosis. JNJ-26481585/Doxorubicin cotreatment leads to caspase activation and caspase-dependent apoptosis, since the broad-range caspase inhibitor N-benzyloxycarbonyl-Val-Ala-Asp-fluoromethylketone (zVAD.fmk) rescues cells from apoptosis. In conclusion, the second-generation HDACI JNJ-26481585 cooperates with chemotherapeutics to engage mitochondrial apoptosis in RMS cells, demonstrating that JNJ-26481585 represents a promising strategy for chemosensitization of RMS.

## INTRODUCTION

RMS is one of the most common soft-tissue sarcomas in children, which comprises two major subtypes, i.e. alveolar rhabdomyosarcoma (ARMS) and embryonal rhabdomyosarcoma (ERMS) [1, 2]. Despite multimodal treatment regimens and advances in combination chemotherapy, patients with primary metastatic disease or relapse are largely resistant to current treatment protocols and have a dismal chance for cure of less than 20% [3]. This highlights the high medical need to develop new treatment regimens in RMS to improve the poor prognosis of these patients. Since conventional therapies with common chemotherapeutics have already been optimized in recent years, new experimental therapies seem to be necessary to improve the outcome of RMS patients, especially with high-risk disease.

Histone deacetylases (HDACs) determine the acetylation status of histones, thereby affecting chromatin topology and gene expression [4]. In addition to histones, HDACs are also able to interact and deacetylate non-histone proteins, thereby affecting their activity, localization or interaction with other proteins [4]. Since HDACs have been shown to be upregulated in many cancer entities, they are considered to represent potential targets for treatment strategies [5, 6]. As a relatively new class of anticancer agents, HDACIs have been shown to induce apoptosis in a variety of cancer cells [7].

HDACIs are able to engage cell death mainly via the two well-described apoptotic pathways, i.e. the extrinsic (death receptor) pathway and the intrinsic (mitochondrial) pathway, which both result in the activation of caspases [8]. Mitochondrial outer membrane permeabilization (MOMP) constitutes a central event in the mitochondrial pathway of apoptosis and is tightly controlled by several pro- and antiapoptotic proteins of the Bcl-2 family [9]. This includes on the one side antiapoptotic proteins (e.g. Bcl-2, Bcl-x_L_ and Mcl-1) and on the other side proapoptotic members such as BH3-only proteins (e.g. Bim, Noxa and Bmf) and the multidomain proteins Bax and Bak [10]. MOMP involves activation of Bax and Bak as pore-forming proteins in the mitochondrial membranes, which results in the release of proteins from the mitochondrial intermembrane space including cytochrome c. In the cytosol, cytochrome c promotes the formation of the apoptosome leading to activation of caspases and apoptotic cell death [9].

JNJ-26481585 (Quisinostat) is a new second-generation, pyrimidyl-hydroxamic acid HDACI, which has a high potency towards class I and II HDACs [11]. JNJ-26481585 showed antitumor activity in patients with advanced solid tumors [11, 12] and in mouse models of multiple myeloma with almost complete reduction of tumor burden [13]. JNJ-26481585 has been documented by the Pediatric Preclinical Testing Program to exhibit improved efficiency compared to common HDACIs, e.g. SAHA (Vorinostat), underscoring that it represents a promising new agent for the treatment of childhood cancers including RMS [14]. Since chemotherapy is a central pillar of current treatment protocols for RMS, anticancer drugs are prime candidates for rational combinations with JNJ-26481585. However, so far it is not known whether or not JNJ-26481585 can be used to increase chemosensitivity of RMS cells. Therefore, the current study aims at investigating the question whether JNJ-26481585 sensitizes RMS cells for chemotherapy-induced cell death and at understanding the underlying molecular mechanisms of action.

## RESULTS

### JNJ-26481585 synergizes with clinically used chemotherapeutic drugs to induce apoptosis in RMS cells

**Figure 1:**
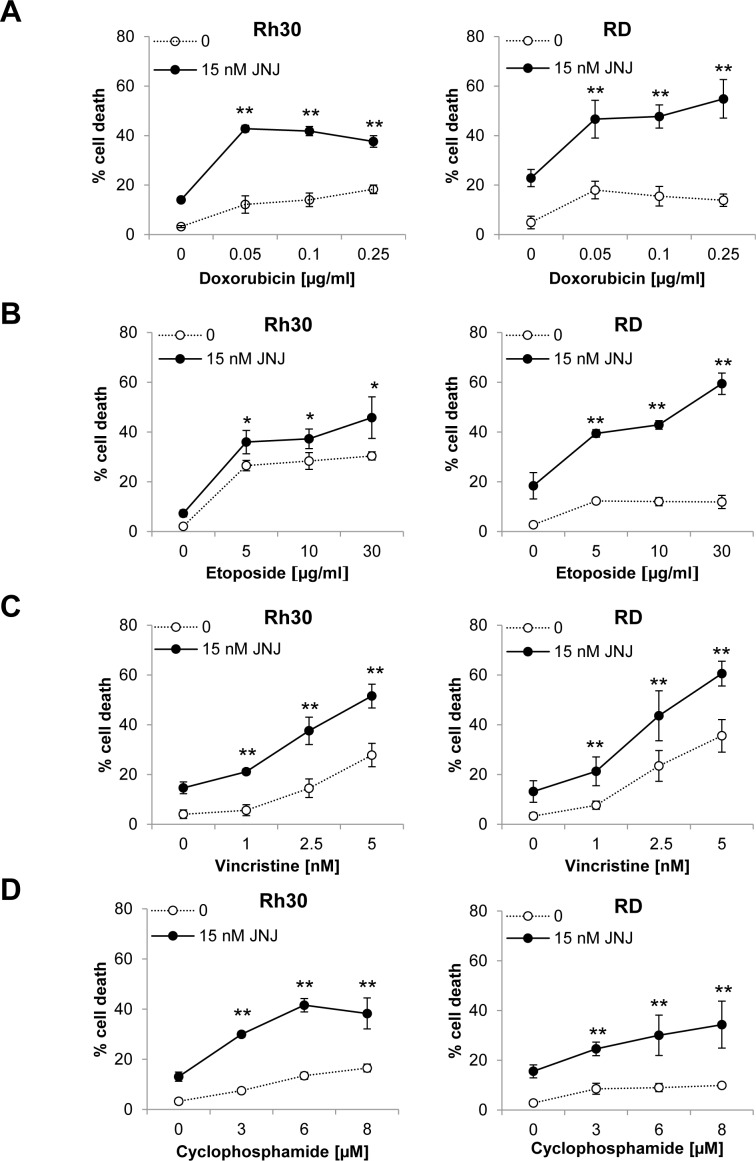

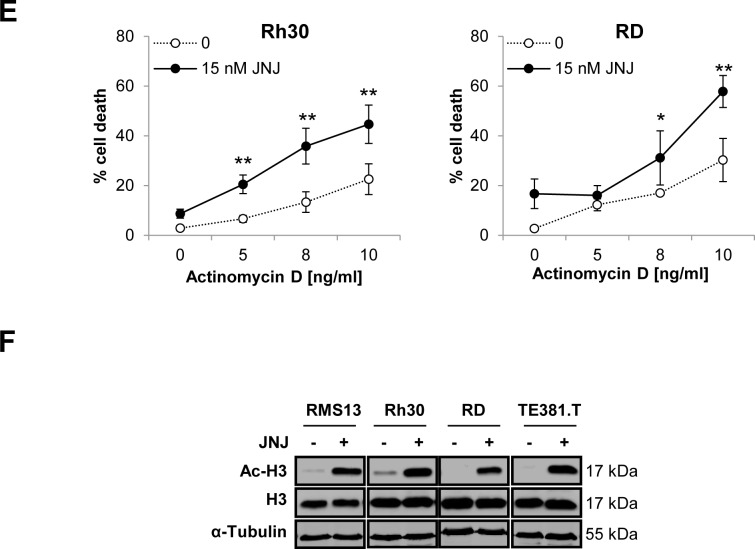
JNJ-26481585 synergizes with clinically used chemotherapeutic drugs to induce apoptosis in RMS cells **A.**-**E.**, Cells were treated with 15 nM JNJ-26481585 and/or indicated concentrations of Doxorubicin **A.**, Etoposide **B.**, Vincristine **C.**, Cyclophosphamide **D.** or Actinomycin D **E.** for 48 hours **A.**-**D.** or 72 hours **E.** Apoptosis was determined by analysis of DNA fragmentation of PI-stained nuclei using flow cytometry. Mean and SD of three experiments performed in triplicate are shown; *P < 0.05, **P < 0.01. **F.**, Cells were treated with 15 nM JNJ-26481585 for 6 hours. Protein expression of acetylated histone H3 (Ac-H3), H3 and α-Tubulin was assessed by Western blotting.

To address the question whether JNJ-26481585 can increase chemosensitivity of RMS cells, we tested the effects of JNJ-26481585 alone and together with chemotherapeutic drugs that are commonly used in clinical trial protocols for the treatment of RMS [15]. We assessed apoptosis by analysis of DNA fragmentation as a characteristic marker of apoptotic cell death in RMS cell lines comprising both the ERMS (RD, TE381.T) and ARMS (Rh30, RMS13) subtypes. Importantly, suboptimal concentrations of JNJ-26481585 synergized with various anticancer drugs, including the topoisomerase II inhibitors Doxorubicin and Etoposide, the vinca alkaloid Vincristine, the alkylating agent Cyclophosphamide and the cytotoxic antibiotic Actinomycin D, to trigger apoptosis in RMS cells as confirmed by calculation of (CI), while treatment with JNJ-26481585 alone induced apoptosis in a dose-dependent manner (Figure 1A-1E, suppl. Figure 1). Concomitant analysis of the acetylation status of histone H3 confirmed that JNJ-26481585 increased histone H3 acetylation under these conditions (Figure 1F). Compared to RMS cells, we observed no additive cytotoxicity of JNJ-26481585 together with Doxorubicin or Etoposide against fibroblasts (Suppl. Figure 2), pointing to some tumor selectivity.

We additionally analyzed SYTOX^®^ Blue-stained nuclei in Rh30 cells as another marker of cell death. Combined treatment or Rh30 cells with JNJ-26481585 and Doxorubicin significantly increased the percentage of SYTOX^®^ Blue-positive cells (Suppl. Figure 3A). Pharmacological rescue experiments showed that addition of the broad-range caspase inhibitor zVAD.fmk, but not the receptor-interacting protein kinase 1 (RIPK1) inhibitor Necrostatin-1s significantly inhibited JNJ-26481585/Doxorubicin-induced cell death (Suppl. Figure 3B), underscoring that JNJ-26481585/Doxorubicin cotreatment triggers apoptotic cell death. This set of experiments demonstrates that JNJ-26481585 sensitizes RMS cells for several clinically used chemotherapeutic agents.

### JNJ-26481585/Doxorubicin cotreatment suppresses clonogenicity of RMS cells and reduces tumor growth *in vivo*

**Figure 2:**
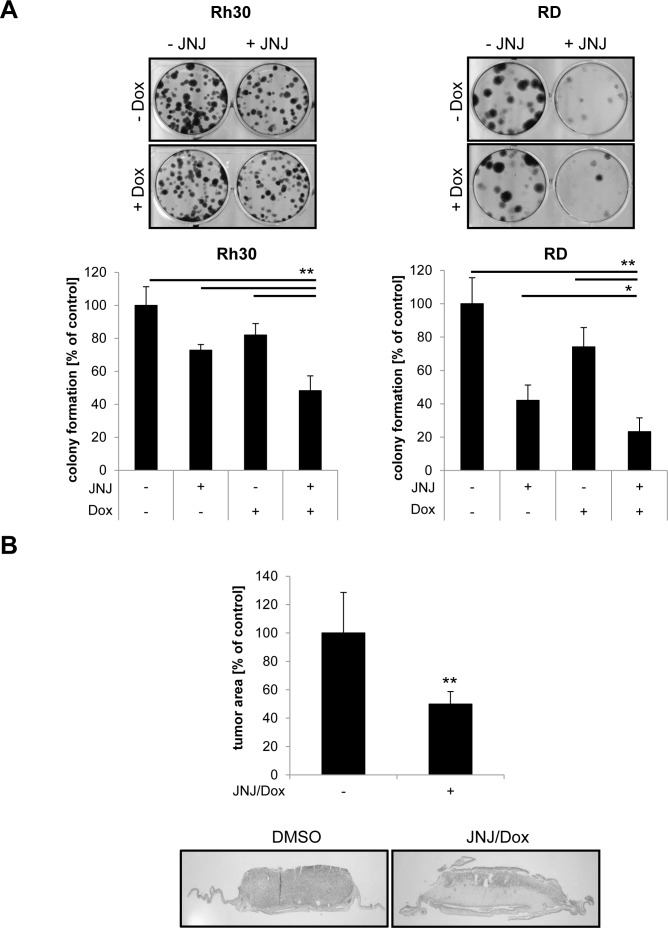
JNJ-26481585/Doxorubicin cotreatment suppresses clonogenicity of RMS cells and reduces tumor growth *in vivo* **A.**, Cells were treated with 7.5 nM of JNJ-26481585 for 23 hours before 0.015 μg/ml Doxorubicin was added for 1 hour. Colony formation was assessed after 12 days as described in Material and Methods. The number of colonies is expressed as percentage of solvent-treated controls (lower panels) and representative images are shown (upper panels). Mean and SEM of at least three independent experiments carried out in duplicate are shown. **B.**, RD cells were seeded on the CAM of fertilized chicken eggs, treated with 15 nM JNJ-26481585 and 0.25 μg/ml Doxorubicin for three days and tumor growth was analyzed using hematoxylin and eosin (H/E)-stained paraffin sections of the CAM. Representative pictures and quantification of tumor area of at least 17 tumors are shown. Mean and SEM of three independent experiments are shown; **P < 0.01.

After this initial screening for synergistic drug interactions, we focused our subsequent studies on JNJ-26481585/Doxorubicin cotreatment, since this combination turned out to be very potent and since Doxorubicin is often clinically used. To explore whether the cotreatment with JNJ-26481585/Doxorubicin has an influence on long-term clonogenic survival of RMS cells, we performed colony assays. Notably, the combination of JNJ-26481585 and Doxorubicin significantly reduced colony formation of RMS cells compared to treatment with either agent alone (Figure 2A).

To assess the antitumor activity of JNJ-26481585/Doxorubicin cotreatment against RMS *in vivo*, we used the chorioallantoic membrane (CAM) model that has successfully been employed in the past to test the anticancer effects of experimental compounds [16-18] and that proved to represent a suitable preclinical RMS model yielding comparable results to xenograft mouse studies [19]. In this model, we implanted RMS cells onto the CAM of chicken embryos, allowed the cells to form tumors and treated them with JNJ-26481585 and Doxorubicin on three consecutive days. On day four, tumors together with the surrounding CAM were sampled for assessment of tumor growth. Importantly, cotreatment with JNJ-26481585/Doxorubicin significantly reduced tumor growth (Figure 2B). Together, these findings show that JNJ-26481585/Doxorubicin cotreatment suppresses clonogenic survival and tumor growth *in vivo* in preclinical RMS models.

### JNJ-26481585 and Doxorubicin cooperate to induce caspase activation and caspase-dependent apoptosis

**Figure 3:**
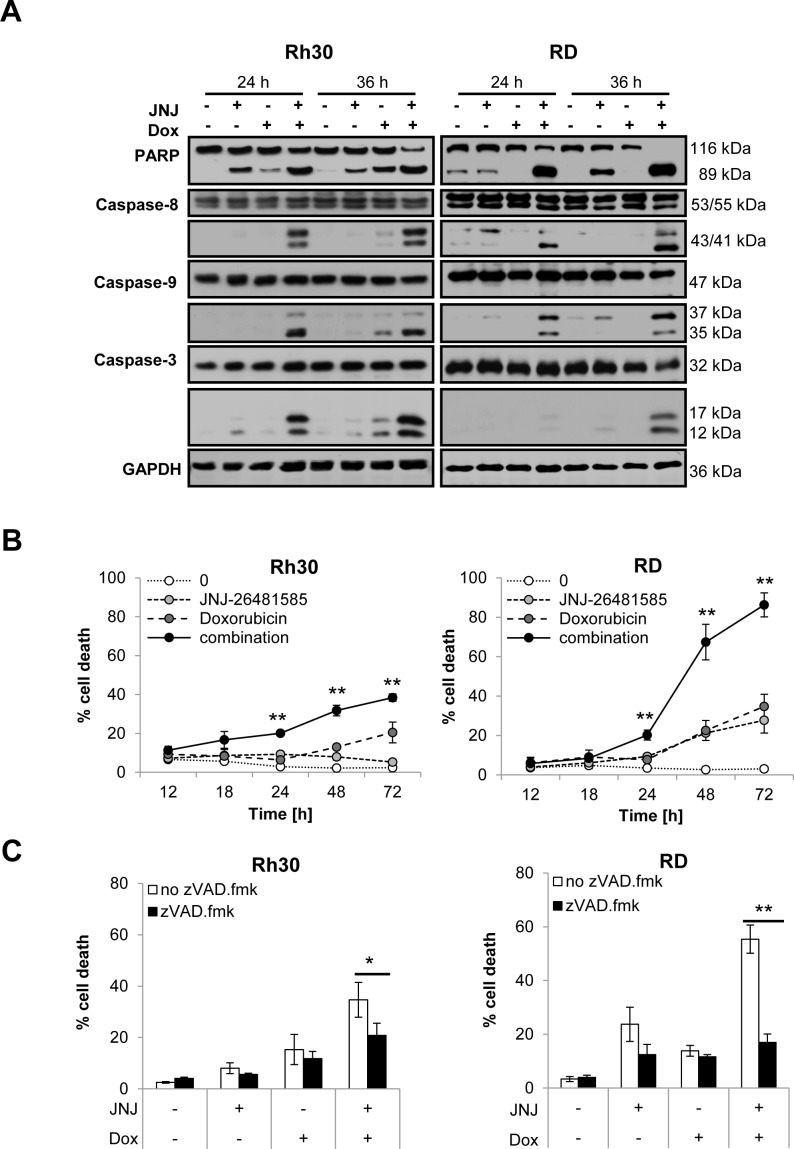

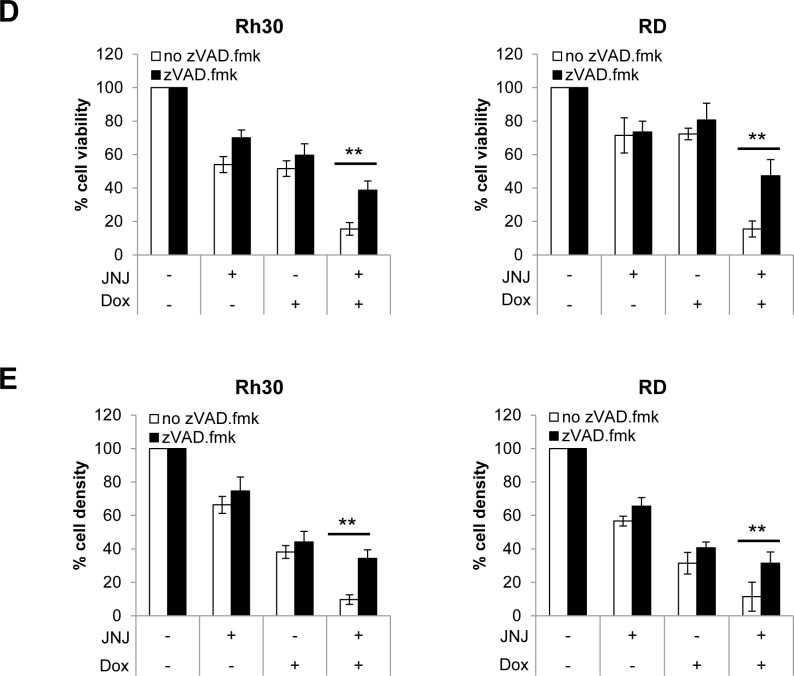
JNJ-26481585 and Doxorubicin cooperate to induce caspase activation and caspase-dependent apoptosis **A.**, Cells were treated with 15 nM JNJ-26481585 and/or 0.25 μg/ml Doxorubicin for indicated times. Cleavage of caspase-9, -8, -3 and PARP was assessed by Western blotting, GAPDH served as loading control. **B.**, Cells were treated for indicated times with 15 nM JNJ-26481585 and/or 0.25 μg/ml Doxorubicin. Apoptosis was determined by analysis of DNA fragmentation of PI-stained nuclei using flow cytometry. Mean and SEM of at least three independent experiments carried out in triplicate are shown; **P < 0.01. **C.**, Cells were treated for 48 hours with 15 nM JNJ-26481585 and/ or 0.25 μg/ml Doxorubicin in the presence and absence of 50 μM zVAD.fmk. Apoptosis was determined by analysis of DNA fragmentation of PI-stained nuclei using flow cytometry. Mean and SEM of at least three independent experiments carried out in triplicate are shown; *P < 0.05, **P < 0.01. **D.**, Cells were treated for 48 hours with 15 nM JNJ-26481585 and/ or 0.25 μg/ml Doxorubicin in the presence and absence of 50 μM zVAD.fmk. Cell viability was measured by MTT assay. Mean and SEM of at least three independent experiments carried out in triplicate are shown; **P < 0.01. **E.**, Cells were treated for 48 hours with 15 nM JNJ-26481585 and/ or 0.25 μg/ml Doxorubicin in the presence and absence of 50 μM zVAD.fmk. Cell density was measured by crystal violet assay. Mean and SEM of at least three independent experiments carried out in triplicate are shown; **P < 0.01.

To gain insights into the underlying molecular events of the observed JNJ-26481585-mediated chemosensitization, we monitored activation of caspases known as key mediators of apoptosis. To this end, we analyzed cleavage of caspases into their active fragments by Western blotting. Notably, JNJ-26481585 and Doxorubicin acted together to trigger cleavage of caspase-8 into active p43/p41 fragments, cleavage of caspase-9 into active p37/p35 fragments, cleavage of caspase-3 into active p17/p12 fragments and cleavage of poly ADP ribose polymerase 1 (PARP) into p89 fragment (Figure 3A). A kinetic analysis revealed that this concerted action of JNJ-25481585 and Doxorubicin to trigger caspase activation occurred at the onset of apoptosis and that the rate of apoptosis upon JNJ-26481585/Doxorubicin cotreatment increased in a time-dependent manner (Figure 3B). To test whether caspase activation is required for apoptosis, we compared the ability of JNJ-26481585/Doxorubicin cotreatment to induce apoptosis in the presence and absence of the broad-range caspase inhibitor zVAD.fmk. Importantly, the addition of zVAD.fmk significantly reduced JNJ-26481585/Doxorubicin-mediated apoptosis as assessed by the analysis of DNA fragmentation (Figure 3C). Similarly, this protective effect of zVAD.fmk against JNJ-26481585/Doxorubicin cotreatment was observed when cell viability was determined by two distinct assays, i.e. 3-(4,5-dimethylthiazol-2-yl)-2,5-diphenyltetrazolium bromide (MTT) assay and crystal violet assay, since zVAD.fmk significantly rescued cells from JNJ-26481585/Doxorubicin-imposed loss of cell viability (Figure 3D, 3E). Together, these experiments demonstrate that JNJ-26481585 and Doxorubicin act in concert to induce caspase activation and caspase-dependent apoptosis.

### JNJ-26481585/Doxorubicin cotreatment shifts the balance of pro- and antiapoptotic proteins

**Figure 4:**
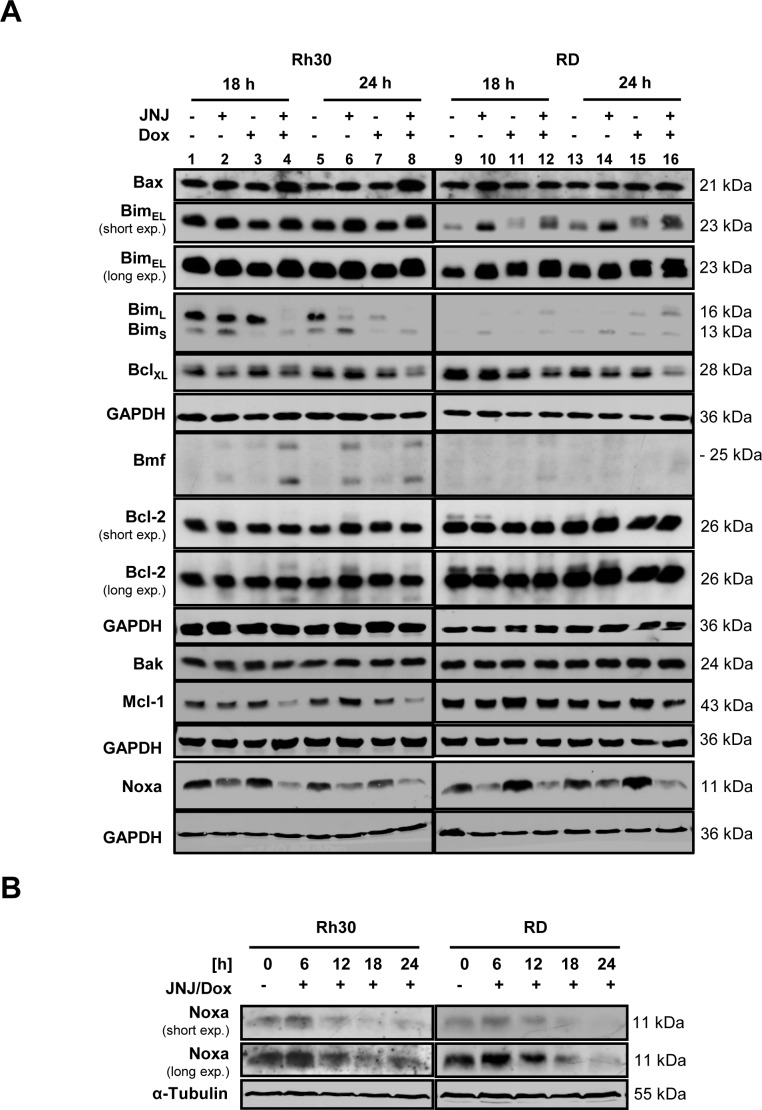

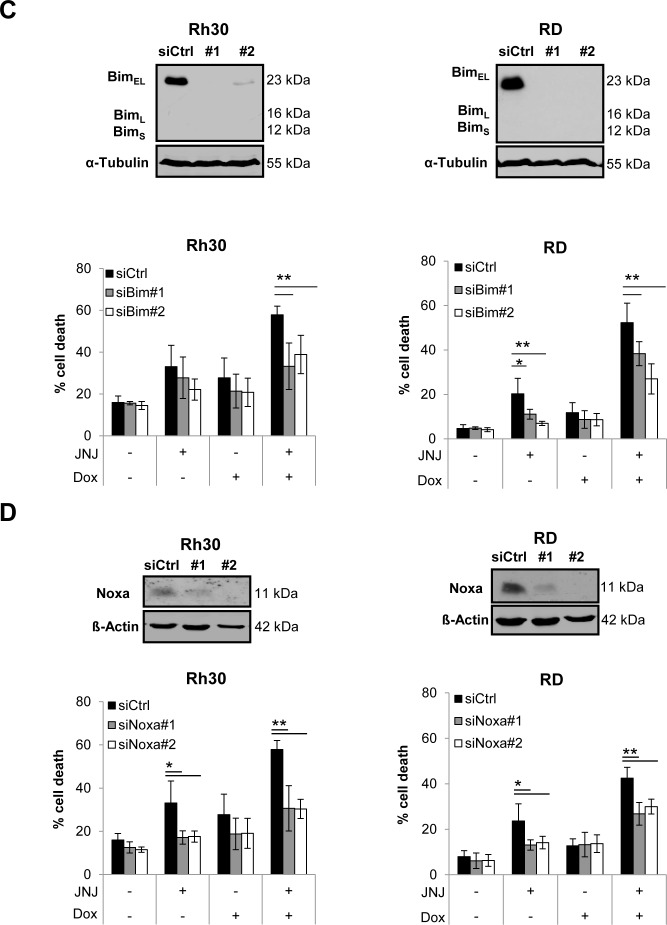
JNJ-26481585/Doxorubicin cotreatment shifts the balance of pro- and antiapoptotic proteins **A.**, Cells were treated with 15 nM JNJ-26481585 and/or 0.25 μg/ml Doxorubicin for indicated times. Protein expression of pro- and antiapoptotic Bcl-2 proteins was assessed by Western blotting, GAPDH served as loading control. **B.**, Cells were treated with 15 nM JNJ-26481585 and/or 0.25 μg/ml Doxorubicin for indicated times. Protein expression of Noxa was assessed by Western blotting, α-Tubulin served as loading control. **C.** and **D.**, RMS cells were transiently transfected with siRNA against Bim, Noxa or non-targeting control siRNA. Expression of Bim and Noxa were assessed by Western blotting, GAPDH or Δ-Actin served as loading controls (upper panels). Transfected cells were treated with 15 nM JNJ-26481585 and/or 0.25 μg/ml Doxorubicin for 30 hours and apoptosis was determined by analysis of DNA fragmentation of PI- or SYTOX^®^ Blue-stained nuclei using flow cytometry (lower panels). Mean and SEM of three independent experiments carried out in triplicate are shown; *P < 0.05; **P < 0.01.

Next, we investigated the effect of JNJ-26481585 and Doxorubicin on expression levels of pro- and antiapoptotic proteins of the Bcl-2 family, which are known as key regulators of apoptosis. Treatment with JNJ-26481585- or JNJ-26481585/ Doxorubicin resulted in upregulation of Bim_EL_ and of Bmf in Rh30 cells (Figure 4A). In addition, Noxa expression increased at early time points upon cotreatment with JNJ-26481585 and Doxorubicin (Figure 4B). Also, JNJ-26481585 and Doxorubicin cooperated to reduce protein levels of Mcl-1 and Bcl-x_L_ (Figure 4A). These findings were confirmed in another RMS cell line (Suppl. Figure 4) and point to a shift in the ratio of pro- and antiapoptotic Bcl-2 proteins upon JNJ-264815/Doxorubicin cotreatment.

To investigate the relevance of Bim and Noxa in JNJ-26481585/Doxorubicin-mediated cell death, we genetically silenced each gene using two distinct siRNA oligonucleotides. Importantly, individual silencing of Bim or Noxa, which was demonstrated by Western blot analysis (Figure 4C and 4D, upper panels), significantly impeded JNJ-26481585/Doxorubicin-induced cell death (Figure 4C and 4D, lower panels). Together, these results demonstrate that the BH3-only proteins Bim and Noxa are necessary for JNJ-26481585-induced cell death.

### JNJ-26481585/Doxorubicin cotreatment stimulates activation of Bax and Bak, which are required for apoptosis

**Figure 5:**
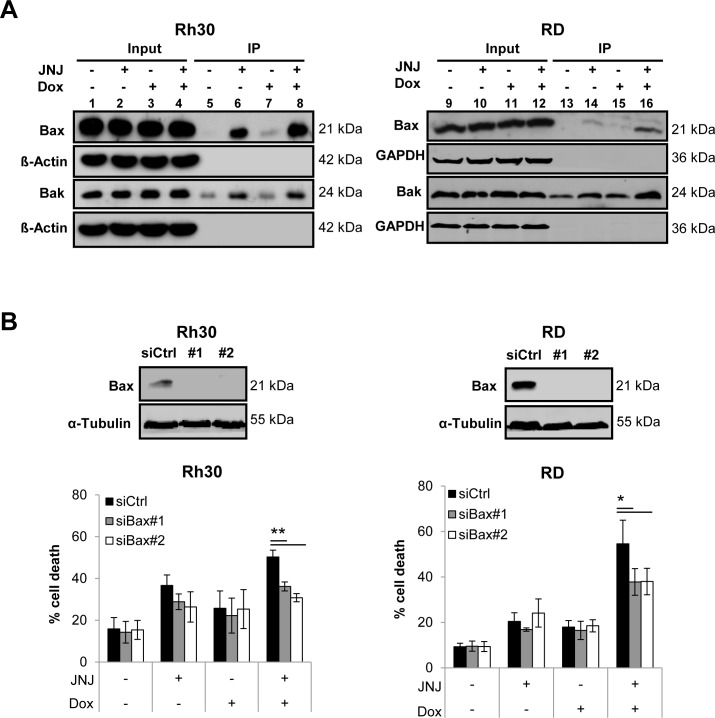

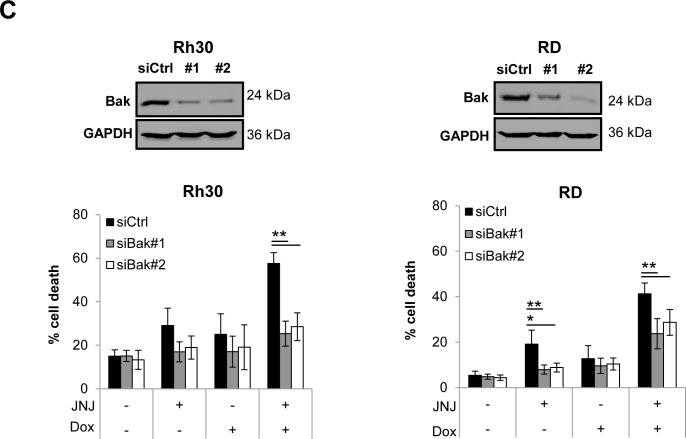
JNJ-26481585/Doxorubicin cotreatment stimulates activation of Bax and Bak, which are required for apoptosis **A.**, Cells were treated for 18 hours with 15 nM JNJ-26481585 and/or 0.25 μg/ml Doxorubicin. Activation of Bax and Bak was assessed by immunoprecipitation using active conformation-specific anti-Bax or anti-Bak antibodies and protein expression of Bax, Bak GAPDH and β-Actin was analyzed by Western blotting. **B.** and **C.**, RMS cells were transiently transfected with siRNA against Bak, Bax or non-targeting control siRNA. Expression of Bak and Bax was assessed by Western blotting, GAPDH or α-Tubulin served as loading controls (upper panels). Transfected cells were treated with 15 nM JNJ-26481585 and/or 0.25 μg/ml Doxorubicin for 30 hours and apoptosis was determined by analysis of DNA fragmentation of PI- or SYTOX^®^ Blue-stained nuclei using flow cytometry (lower panels). Mean and SEM of three independent experiments carried out in triplicate are shown; *P < 0.05; **P < 0.01.

Since we observed that expression levels of Bax and Bak remained largely unchanged upon treatment with JNJ-26481585 and Doxorubicin in both cell lines (Figure 4A), we asked the question whether JNJ-26481585/Doxorubicin cotreatment instead stimulates activation of Bax and Bak, two multidomain proapoptotic Bcl-2 proteins that play an important role in the control of MOMP. To address this question we immunoprecipitated Bax and Bak by conformation-specific antibodies, since a conformational change marks their activation status [20, 21]. Of note, JNJ-26481585 and Doxorubicin cooperated to stimulate activation of Bax and/or Bak (Figure 5A, lanes 6, 8, 14 and 16).

To investigate the functional relevance of Bax/Bak activation for JNJ-26481585/Doxorubicin-mediated apoptosis we genetically silenced each gene using two distinct siRNA oligonucleotides (Figure 5B and 5C, upper panels). Remarkably, knockdown of either Bax or Bak significantly reduced JNJ-26481585/Doxorubicin-induced apoptosis (Figure 5B and 5C, lower panels). This shows that Bax/Bak activation is required for JNJ-26481585/Doxorubicin-induced cell death.

### Overexpression of Bcl-2 inhibits JNJ-26481585/Doxorubicin-induced apoptosis

**Figure 6:**
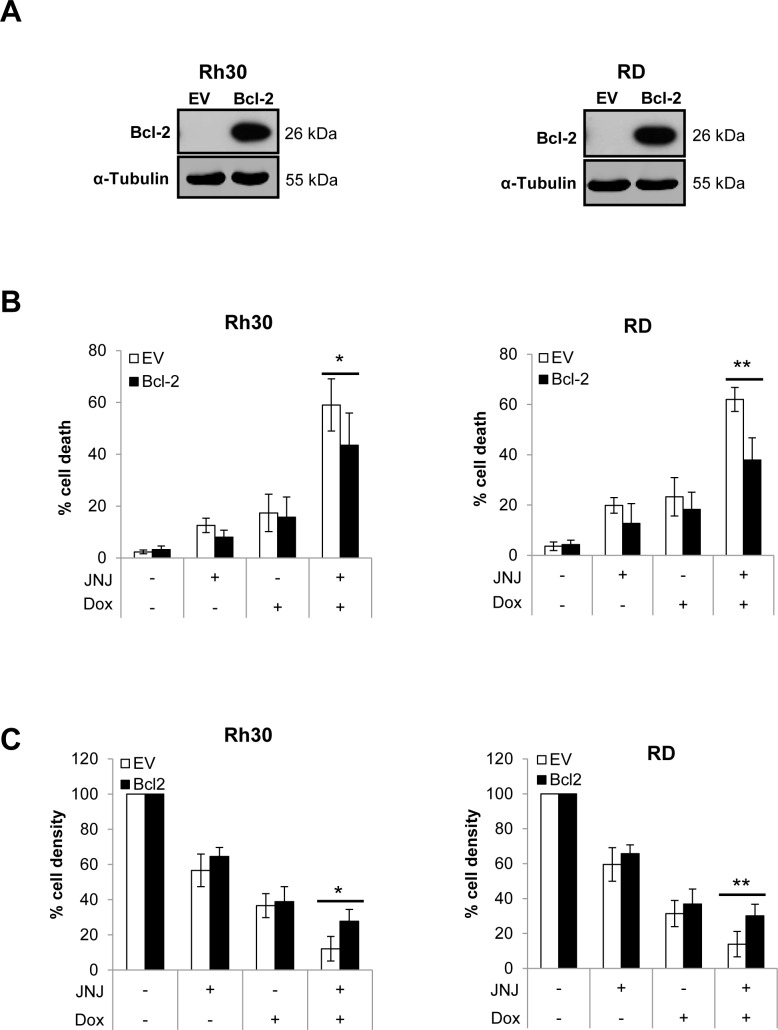
Overexpression of Bcl-2 inhibits JNJ-26481585/Doxorubicin-induced apoptosis **A.**, Cells were transfected with murine Bcl-2 or empty vector (EV) and expression of Bcl-2 was assessed by Western blotting, α-Tubulin served as loading control. **B.**, Cells were treated with 15 nM JNJ-26481585 and/or 0.25 μg/ml Doxorubicin for 48 hours. Apoptosis was determined by analysis of DNA fragmentation of PI-stained nuclei using flow cytometry. Mean and SEM of three independent experiments carried out in triplicate are shown; *P < 0.05; **P < 0.01. **C.**, Cells were treated with 15 nM JNJ-26481585 and/or 0.25 μg/ml Doxorubicin for 48 hours. Cell density was measured by crystal violet assay. Mean and SEM of at least three independent experiments carried out in triplicate are shown; *P < 0.05, **P < 0.01.

To further explore whether activation of the mitochondrial apoptotic pathway represents a critical event during JNJ-26481585/Doxorubicin-induced apoptosis, we overexpressed the antiapoptotic protein Bcl-2, which has been reported to impair mitochondria-mediated apoptotic signaling events [9]. Ectopic expression of murine Bcl-2 was confirmed using an antibody that specifically detects the ectopically expressed murine Bcl-2 (Figure 6A). Importantly, overexpression of Bcl-2 significantly reduced JNJ-26481585/Doxorubicin-induced DNA fragmentation as well as loss of cell viability (Figure 6B and 6C). Also, Bcl-2 overexpression abolished JNJ-26481585/Doxorubicin-stimulated activation of Bax and Bak (Suppl. Figure 5). These results emphasize the notion that JNJ-26481585/Doxorubicin-induced apoptosis depends on intact signaling via the mitochondrial pathway of apoptosis.

## DISCUSSION

In recent years, there has been a growing interest in using HDACIs for the treatment of cancers including pediatric malignancies such as RMS. Of note, HDACIs as a class turned out to be active against RMS cells in a recent screen of a custom library of about 200 compounds including chemotherapeutics, Food and Drug Administration-approved drugs and small molecules with biological activity [22]. Furthermore, Vorinostat is currently being evaluated in early clinical trials for the treatment of childhood cancers including RMS [23]. The second-generation HDACI JNJ-26481585 has been documented to exhibit improved pharmacodynamic properties over other HDAC inhibitory compounds, demonstrating sustained histone acetylation in tissues and tumors [11]. Against this background, we investigated the question whether JNJ-26481585 could be employed to enhance the sensitivity of RMS cells towards chemotherapeutics.

In this study, we identify the synergistic interaction of JNJ-26481585 together with chemotherapeutic agents to trigger apoptosis in RMS cells. JNJ-26481585 sensitizes RMS cells for apoptosis induced by several anticancer drugs, which are commonly used in the clinic for the treatment of RMS, including Doxorubicin, Etoposide, Vincristine, Cyclophosphamide and Actinomycin D [15], thus underscoring the clinical relevance of this chemosensitization. In contrast to RMS cells, JNJ-26481585 does not enhance the cytotoxicity of anticancer drugs such as Doxorubicin or Etoposide against non-malignant fibroblasts, pointing to some tumor selectivity. Importantly, the combination of JNJ-26481585 and Doxorubicin significantly suppresses tumor growth in a preclinical *in vivo* model of RMS and inhibits long-term clonogenic growth of RMS cells. While we used co-treatment with JNJ-26481585 and Doxorubicin to identify synergistic drug interactions, it remains subject to further studies to explore the effects of different treatment schedules on synergism.

Mechanistic studies revealed that the synergism of JNJ-26481585 and Doxorubicin involves cooperative induction of apoptosis via the mitochondrial pathway, as demonstrated by several lines of evidence. First, activation of the mitochondrial apoptotic pathway is demonstrated by the following typical parameters. JNJ-26481585 and Doxorubicin act in concert to shift the ratio of pro- and antiapoptotic Bcl-2 proteins in favor of apoptosis, thereby facilitating the activation of the mitochondrial gatekeeper proteins Bax and Bak, which in turn leads to activation of caspases and DNA fragmentation. Caspase-dependent apoptosis was confirmed by experiments using the broad-range caspase inhibitor zVAD.fmk, which significantly rescues JNJ-26481585/Doxorubicin-induced apoptosis. Second, rescue experiments demonstrate that the BH3-only domain proteins Bim and Noxa as well as the multidomain proteins Bax and Bak are required for apoptosis, since individual silencing of Bim, Noxa, Bax or Bak significantly impedes JNJ-26481585/Doxorubicin-induced apoptosis. Third, JNJ-26481585/Doxorubicin cotreatment causes downregulation of the antiapoptotic proteins Mcl-1 and Bcl-x_L_, which is in line with previous reports showing that HDACIs can also downregulate gene expression levels [8]. Fourth, ectopic expression of Bcl-2, which is known to impede mitochondria-mediated apoptotic signaling events, significantly rescues JNJ-26481585/Doxorubicin-induced apoptosis. Together, these mechanistic studies emphasize the critical role of the mitochondrial apoptosis pathway for JNJ-26481585/Doxorubicin-induced apoptosis. In line with this notion, JNJ-26481585 has previously been described to act by downregulation of Mcl-1 and upregulation of Bid and Bim cells in multiple myeloma cells [24].

It has been shown before that HDACIs predominantly act by inducing apoptosis, either by the extrinsic or the intrinsic apoptotic pathway depending on the context [8]. We recently reported that Vorinostat, a first-generation HDACI, is able to sensitize RMS and Ewing sarcoma towards classical chemotherapeutics, especially Doxorubicin and Etoposide [25, 26]. However, nothing is known so far on rational combination therapies using the second-generation HDACI JNJ-26481585 in RMS. JNJ-26481585 represents a promising compound to target HDACs in pediatric cancers, since it has been reported to retard tumor growth in the majority of solid pediatric xenografts studied [14], whereas Vorinostat induced no objective responses among 30 evaluable solid childhood tumor xenografts [27]. Our study demonstrates for the very first time that JNJ-26481585 primes RMS cells for anticancer drug-triggered apoptosis. Since chemotherapy represents a central element of current treatment protocols for RMS [15], this synergistic interaction is of special interest for future clinical translation. Thus, this study provides an important rationale to transfer combinations of JNJ-26481585 together with anticancer drugs into clinical application for the treatment of RMS.

## MATERIALS AND METHODS

### Cell culture and chemicals

RMS cell lines were obtained from the American Type Culture Collection (Manassas, VA, USA) and maintained in RPMI 1640 or DMEM medium (Life Technologies, Inc., Darmstadt, Germany), supplemented with 10% fetal calf serum (FCS) (Biochrom, Berlin, Germany), 1% penicillin/streptomycin and 1 mM Sodium Pyruvate (Invitrogen, Karlsruhe, Germany). zVAD.fmk was purchased from Bachem (Heidelberg, Germany), 5-((7-Cl-1H-indol-3-yl)methyl)-3-methylimidazolidine-2,4-dione) (7-Cl-O-Nec-1, Nec-1s) from Merck (Darmstadt, Germany), JNJ-26481585 from Selleck Chemicals (Houston, TX, USA), and all other chemicals from Sigma (Deisenhofen, Germany) unless indicated otherwise.

### Determination of cell viability, apoptosis and clonogenic growth

For determination of cell viability and apoptosis cells were seeded at 0.3 × 105 cells/cm^2^. Cell viability was determined by MTT assay according to the manufacturer's instructions (Roche Diagnostics, Mannheim, Germany) or by crystal violet assay by staining viable cells with crystal violet solution (0.5% crystal violet, 30% ethanol, and 3% formaldehyde). Apoptosis was determined by flow cytometric analysis (FACSCanto II, BD Biosciences, Heidelberg, Germany) of DNA fragmentation of propidium iodide (PI)-stained nuclei as described previously [28]. Briefly, cells were seeded in a 24-well plate, allowed to settle overnight and treated with indicated drug concentrations. After indicated treatment times, cells were washed with ice-cold PBS, resuspended in a buffer containing 0.05% trisodium citrate dihydrate pH 7.4, 0.05% Triton X-100, 50 μg/ml PI), incubated at 4°C and apoptosis was determined by flow cytometry. In addition, apoptosis was assessed by SYTOX® Blue-stained nuclei according to the manufacturer's instructions (Life Technologies, Inc.). To determine colony formation, 200 cells were seeded in a 6-well tissue culture plate, allowed to settle overnight and treated with 7.5 nM JNJ-26481585 for 23 hours before 0.015 μg/ml Doxorubicin was added for 1 hour. Then, medium was exchanged and colonies were stained after 12 days with crystal violet solution. To analyze cooperative drug interaction, drug concentrations and treatment duration were modified for colony formation assay due to the prolonged assay period and the reduced initial cell density. Colonies containing more than 50 cells were counted and the percentage of surviving colonies relative to solvent-treated controls was calculated.

### Transduction

For Bcl-2 overexpression, Phoenix packaging cells were transfected with 20 μg of murine stem-cell virus (pMSCV, Clontech, Mountain View CA) vector containing murine Bcl-2 or empty vector using calcium phosphate transfection as described previously [25]. Stable cell lines were generated by lentiviral transduction and were selected with 10 μg/ml Blasticidin (Invitrogen).

### RNA interference

For transient knockdown by siRNA, cells were reversely transfected with 10-20 nM SilencerSelect siRNA (Life Technologies, Inc.), i.e. control siRNA (4390843) or targeting siRNAs (s1880 and s1881 for Bak, s1888 and s1890 for Bax, s195011 and s223065 for Bim, s10708 and s10709 for Noxa) using Lipofectamine RNAi Max reagent and OptiMEM (Life Technologies, Inc.).

### Western blot analysis

Western blot analysis was performed as described previously [28] using the following antibodies: mouse anti-caspase-8, mouse anti-Noxa, rat anti-Bmf (Alexis Biochemicals, Grünberg, Germany), mouse anti-Bcl-2, rabbit anti-Bcl-x_L_, mouse anti-Bax, rabbit anti-Bak (BD Transduction Laboratories, Heidelberg, Germany), rabbit anti-caspase-3, rabbit anti-caspase-9, rabbit anti-Bim, mouse anti-PARP (Cell Signaling, Beverly, MA), acetylated histone H3 (Upstate Biotechnology, Lake Placid, NY), rabbit anti-Mcl-1 (Stressgene, Victoria, BC), rabbit histone H3 (Abcam, Cambridge, UK). Mouse anti-GAPDH (HyTest, Turku, Finland), mouse α-Tubulin (Calbiochem, Darmstadt, Germany) or mouse β-Actin (Sigma) were used as loading controls. Goat anti-mouse IgG, goat anti-rabbit IgG conjugated to horseradish peroxidase (Santa Cruz Biotechnology, Santa Cruz, CA) were used as secondary antibodies. Enhanced chemiluminescence (Amersham Bioscience, Freiburg, Germany) or infrared dye-labeled secondary antibodies and infrared imaging (Odyssey Imaging System, LICOR Bioscience, Bad Homburg, Germany) were used for detection. Representative blots of at least two independent experiments are shown.

### Determination of activation of Bax and Bak

For detection of active Bax or Bak, cells were lysed in CHAPS lysis buffer (10 mM HEPES (pH 7.4); 150 mM NaCl; 1% CHAPS) as previously described [29]. Briefly, 700-1000 μg protein was immunoprecipitated and incubated overnight at 4°C with 2 μg/ml mouse anti-Bax antibody (6A7, Sigma) or anti-Bak antibody (Ab-1; Calbiochem) and 10 μl pan-mouse IgG Dynabeads (Dako, Hamburg, Germany) and washed with CHAPS buffer. The precipitate was analyzed for Bax and Bak expression by Western blotting using the Bax NT antibody (Merck) or Bak antibody (BD Biosciences).

### CAM assay

CAM assay was done as described previously [29]. Briefly, 1 × 106 cells were implanted on the CAM of fertilized chicken eggs on day 8 of incubation. The next three days, cells were treated with 15 nM JNJ-26481585 and 0.25 μg/ml Doxorubicin. Four days after inoculation, tumors were excised with the surrounding CAM, fixed in 4% paraformaldehyde, embedded in paraffin, cut in 3 μm-sections and stained with 1:1 hematoxylin and 0.5% eosin solution for histological analysis. Images were digitally recorded with an AX70 microscope (Olympus, Center Valley, PA, USA) and tumor area was analyzed by ImageJ digital imaging software (NIH, Bethesda, MA, USA).

### Statistical analysis

Statistical significance was assessed by Student's t-Test (two-tailed distribution, two-sample, unequal variance). Drug interaction was analyzed by the CI method using CalcuSyn software (Biosoft, Cambridge, UK). CI <0.9 indicates synergism, 0.9-1.1 additivity and >1.1 antagonism.

## SUPPLEMENTARY MATERIAL FIGURES AND TABLE


