# Supplementary material for: JNJ-26481585 primes rhabdomyosarcoma cells for chemotherapeutics by engaging the mitochondrial pathway of apoptosis
Source: Oncotarget. 2015 Oct 12;6(35):37836–51. doi: 10.18632/oncotarget.6097 (PMC4741969; doi:10.18632/oncotarget.6097)
Supplement: Supplementary file 1 [file oncotarget-06-37836-s001.pdf]

JNJ-26481585 primes rhabdomyosarcoma cells for chemotherapeutics by engaging the mitochondrial pathway of apoptosis

Supplementary Material

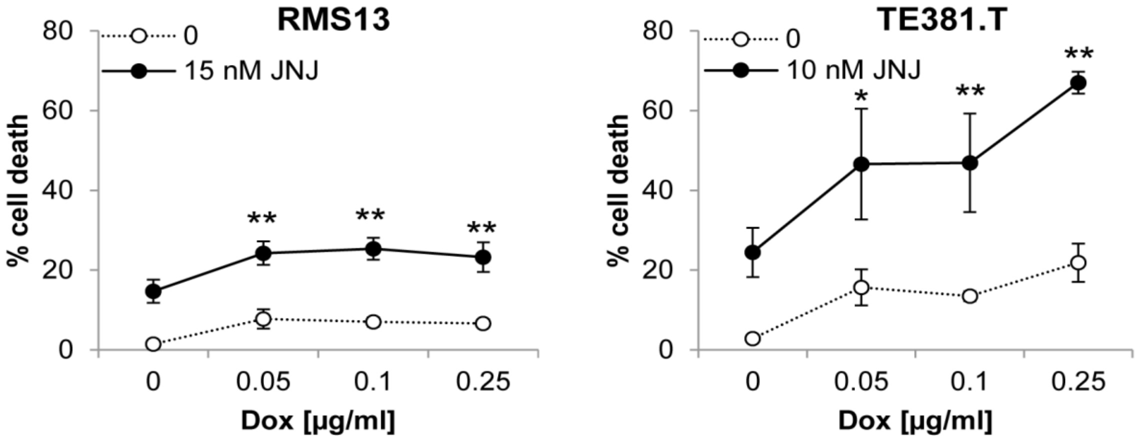

Figure 1: JNJ-26481585 sensitizes RMS cells for chemotherapy-induced apoptosis.
Cells were treated with indicated concentrations of JNJ-26481585 and/or Doxorubicin for 48 hours. Apoptosis was determined by analysis of DNA fragmentation of PI-stained nuclei using flow cytometry. Mean and SD of three experiments performed in triplicate are shown; *P<0.05, **P<0.01.

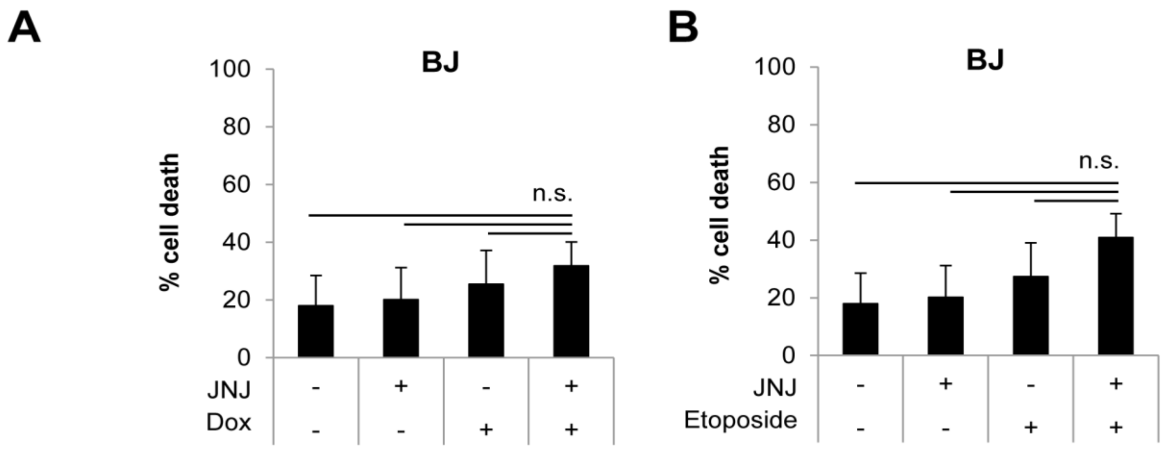

Figure 2: Effect of JNJ-26481585/Doxorubicin and JNJ-26481585/Etoposide cotreatment on BJ fibroblasts.

A, Human BJ fibroblasts were treated with 15 nM of JNJ-26481585 and/or 0.25 µg/ml Doxorubicin for 48 hours. Apoptosis was determined by analysis of DNA fragmentation of PI-stained nuclei using flow cytometry. Mean ± SEM of at least three independent experiments carried out in triplicate are shown. B, Human BJ fibroblasts were treated with 15 nM of JNJ-26481585 and/or 100 µg/ml Etoposide for 48 hours. Apoptosis was determined by analysis of DNA fragmentation of PI-stained nuclei using flow cytometry. Mean ± SEM of at least three independent experiments carried out in triplicate are shown.

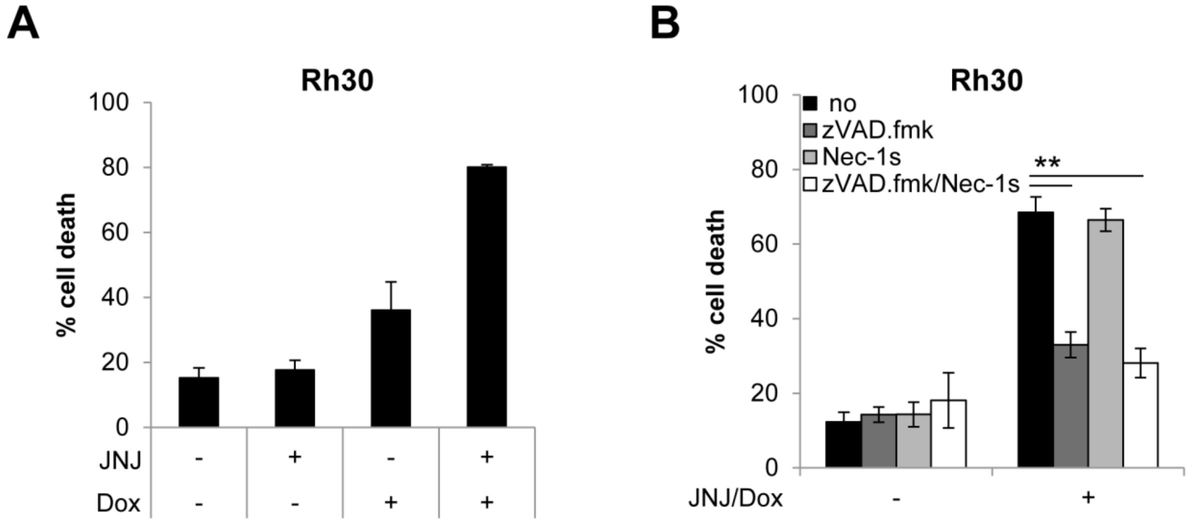

Figure 3: JNJ-26481585/Doxorubicin cotretament trigger caspase-dependent and RIPK1-independent cell death.
A, Rh30 cells were treated for 48 hours with 15 nM JNJ-26481585 and/or 0.25 μg/ml Doxorubicin. Cell death was determined by SYTOX® Blue-stained nuclei using flow cytometry. Mean ± SEM of at least three independent experiments carried out in triplicate are shown. B, Rh30 cells were treated for 48 hours with 15 nM JNJ-26481585 and 0.25 μg/ml Doxorubicin, +/- 50 μM zVAD.fmk and 60 μM Nec-1s. Cell death was determined by SYTOX® Blue-stained nuclei using flow cytometry. Mean ± SEM of at least three independent experiments carried out in triplicate are shown; **$P<0.01$.

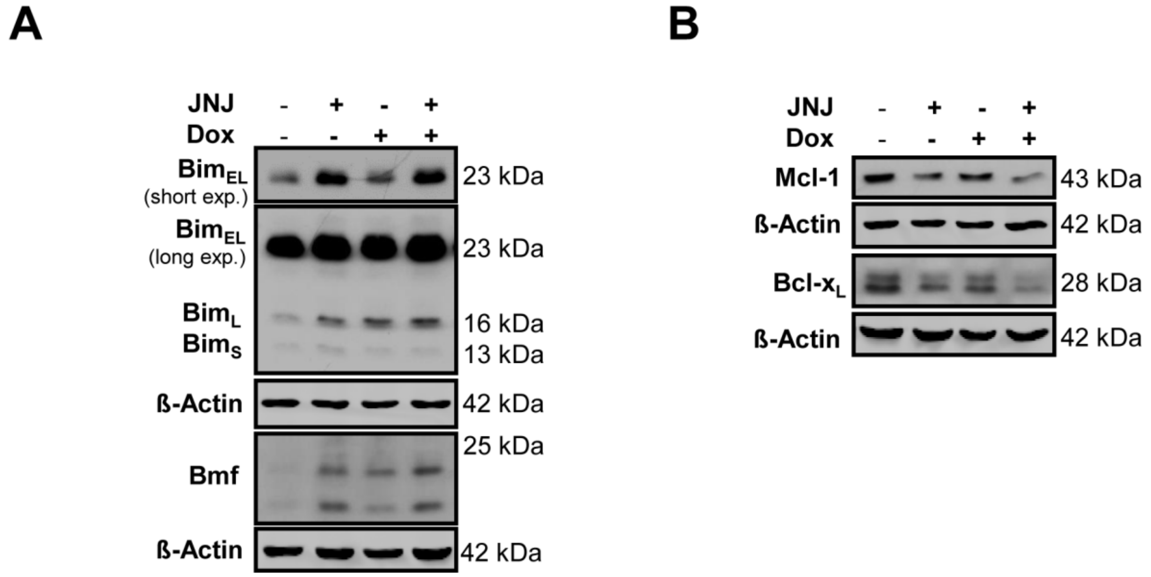

Figure 4: JNJ-26481585/Doxorubicin cotreatment shifts the balance of pro- and antiapoptotic proteins.
A, RMS13 cells were treated with 15 nM JNJ-26481585 and/or 0.25 µg/ml Doxorubicin for 18 hours. Protein expression of Bim and Bmf were assessed by Western blotting, β-Actin served as loading control. B, RMS13 cells were treated with 15 nM JNJ-26481585 and/or 0.25 µg/ml Doxorubicin for 24 hours. Protein expression of Mcl-1 and Bcl-x_L were assessed by Western blotting, β-Actin served served as loading control.

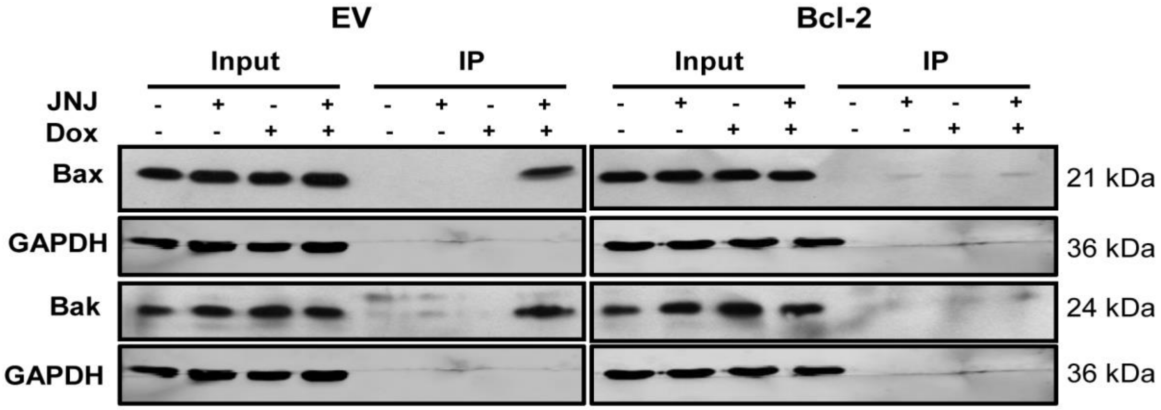

Figure 5: Overexpression of Bcl-2 abrogates JNJ-26481585/Doxorubicin-induced activation of Bax and Bak.

RD cells transfected with murine Bcl-2 or empty vector (EV) were treated with 15 nM JNJ-26481585 and/or 0.25 µg/ml Doxorubicin for 24 hours. Bax and Bak activation were assessed by immunoprecipitation using an active conformation-specific anti-Bax or anti-Bak antibody and expression of Bax, and Bak was analyzed by Western blotting, GAPDH served as loading control.

Suppl. Table. 1: Synergistic induction of apoptosis by JNJ-26481585 and chemotherapeutic drugs.

Rh30	JNJ-26481585 [nM]			TE381.T	JNJ-26481585 [nM]		
**Doxorubicin [µg/ml]**	**10**	**15**	**20**		**10**	**15**	**20**
**0.05**	0.287	0.336	0.439		0.612	0.681	0.744
**0.1**	0.244	0.351	0.477		0.610	0.662	0.724
**0.25**	0.344	0.426	0.524		0.400	0.499	0.600
**Rh30**	**JNJ-26481585 [nM]**			**RD**	**JNJ-26481585 [nM]**		
**Etoposide [µg/ml]**	**10**	**15**	**20**		**10**	**15**	**20**
**5**	0.112	0.162	0.198		0.444	0.513	0.470
**10**	0.097	0.153	0.178		0.430	0.469	0.443
**30**	0.084	0.109	0.136		0.308	0.306	0.293
**Cyclophosphamide [µM]**	**10**	**15**	**20**		**10**	**15**	**20**
**3**	0.626	0.561	0.525		0.737	0.749	0.701
**6**	0.547	0.434	0.554		0.653	0.631	0.548
**8**	0.576	0.556	0.576		0.686	0.556	0.546
**Vincristine [nM]**	**10**	**15**	**20**		**10**	**15**	**20**
**0.5**	1.335	0.943	1.141		1.061	1.276	0.802
**1**	0.854	0.737	0.905		0.653	0.909	0.586
**1.5**	0.832	0.691	1.030		0.677	0.805	0.706

Combination index (CI) was calculated as described in Materials and Methods for apoptosis induced by combined treatment for 48 hours with indicated concentrations of JNJ-26481585 and chemotherapeutic drugs. CI <0.9 indicates synergism, 0.9-1.1 additivity and >1.1 antagonism.
